# Host factors associated with *Giardia duodenalis* infection in dogs across multiple diagnostic tests

**DOI:** 10.1186/s13071-019-3810-3

**Published:** 2019-11-21

**Authors:** Mathilde Uiterwijk, Rolf Nijsse, Frans N. J. Kooyman, Jaap A. Wagenaar, Lapo Mughini-Gras, Harm W. Ploeger

**Affiliations:** 1 Department of Infectious Diseases and Immunology, Faculty of Veterinary Medicine, Utrecht University, Utrecht, The Netherlands; 2 Present Address: The Netherlands Food and Consumer Product Safety Authority (NVWA), Centre Monitoring Vectors, Wageningen, The Netherlands; 3 Wageningen Bioveterinary Research, Lelystad, The Netherlands; 4 National Institute for Public Health and the Environment (RIVM), Centre for Infectious Disease Control (CIb), Bilthoven, The Netherlands; 5 Institute for Risk Assessment Sciences (IRAS), Faculty of Veterinary Medicine, Utrecht University, Utrecht, The Netherlands

**Keywords:** Giardiosis, Canine, Endoparasites, Loose stool, qPCR, IDEXX SNAP^®^*Giardia*, DFA

## Abstract

**Background:**

The aim of this study was to assess potential associations between *Giardia duodenalis* infection in dogs, as determined by three diagnostic tests, and dog’s group of origin, fecal consistency, age, sex, neuter status, and co-infections with other gastrointestinal parasites.

**Methods:**

Fecal samples from 1291 dogs from four groups (household, shelter, hunting and clinical dogs) were tested with qPCR, rapid enzyme immunochromatographic assay (IDEXX SNAP^®^ *Giardia*), and direct immunofluorescence (DFA, Merifluor) for presence of *G. duodenalis*. Moreover, fecal samples were tested with centrifugation sedimentation flotation (CSF) coproscopical analysis for presence of gastrointestinal parasites. Associations were expressed as odds ratios (ORs).

**Results:**

Several significant associations were found, of which a few were consistent for all three tests and *Giardia* positivity in general (positive with at least one of these tests). Dogs older than one year were significantly less likely to test positive for *Giardia* than younger dogs. Group-housed dogs, especially hunting dogs, were significantly more likely to test positive for *Giardia* compared to household and clinical dogs. A consistently significant association with *Trichuris* appeared to be driven by the high prevalence in hunting dogs. Although there was no significant association between loose stool and *Giardia* infection in the overall population, household dogs were significantly more likely to test *Giardia*-positive when having loose stool. Overall, *Giardia*-positive dogs with loose stool shed significantly more cysts, both determined semi-quantitatively with CSF and quantitatively by qPCR, than positive dogs with no loose stool. When other gastrointestinal parasites were present, significantly fewer cysts were detected with CSF, but this was not confirmed with qPCR.

**Conclusion:**

*Giardia* is the most common gastrointestinal parasite in Dutch dogs, except for hunting dogs, in which *Trichuris* and strongyle-type eggs (hookworms) prevailed. *Giardia* infection was not significantly associated with loose stool, except for household dogs. Young dogs and group-housed dogs were significantly more often *Giardia*-positive. These associations were consistent across diagnostic tests. Young dogs, clinical dogs and dogs with loose stool shed *Giardia* cysts in the highest numbers. If another gastrointestinal parasite was present lower numbers of cysts were observed by microscope (CSF), but not with a molecular method (qPCR).

## Introduction

*Giardia duodenalis* (syns. *G. lamblia* and *G. intestinalis*) is a gastrointestinal parasite of mammals with a worldwide distribution. In the small intestine, the trophozoites are either attached to the gut cells with their ventral adhesive disc or free in the lumen. To acquire an environmentally resistant form, the trophozoites develop into cysts, which are immediately infective after shedding in the stool. Since many dogs exhibit coprophagic behavior [1], ingestion of cysts with feces is an important transmission route in canids and contributes to *G. duodenalis* being one of the most reported gastrointestinal parasites in dogs.

Whether ingestion of cysts leads to infection (giardiasis) and subsequently clinical symptoms (giardiosis) depends on factors related to the host (such as co-infections, age, sex, genetic predisposition, immune competence, gut microbiota, nutritional status, stress, etc.) and to the agent (such as assemblage, production of proteolytic enzymes, variant-specific surface proteins, cyst quantity, etc.) [2–7]. Consideration needs to be given to the fact that in many cases, *Giardia* infection remains subclinical [8, 9]. Several studies have shown that the fecal consistency score is comparable between dogs with or without *G. duodenalis* infection [10–12]. Moreover, in children [13–15] and in a mouse model [16], it was shown that *G. duodenalis* infection exerts a protective effect against diarrhea. When co-infections of *G. duodenalis* with other gastrointestinal parasites are considered, negative associations between the presence of *G. duodenalis* and other gastrointestinal parasites are reported in humans [17, 18] and dogs [19]. There is a debate as to whether *Giardia* prevalence has increased in dogs over the years in the western world. If so, it can be hypothesized that standard deworming protocols have led to increased susceptibility towards *G. duodenalis* infections in dogs [11]. This has also been hypothesized in humans [17, 20]. On the other hand, positive associations between the presence of *G. duodenalis* and other gastrointestinal parasites have been reported as well in children [21] and in dogs [22, 23]. In Greek dogs, positive associations were found with *Toxocara canis* and *Trichuris vulpis*, and a negative association with *Isospora* spp. [24].

For diagnosing *Giardia* infections, several assays in veterinary and human medicine are available. The test characteristics, especially sensitivity and specificity, vary [25] and influence reported prevalence and associations. Using a large number of dogs from different groups, the aim of this study was to assess the associations between the presence of *G. duodenalis* and fecal consistency, dog group, age, sex, neuter status and co-infection with other gastrointestinal parasites. For *Giardia* detection, three different diagnostic tests (qPCR, IDEXX SNAP^®^ *Giardia* and DFA) were used as to allow for the assessment of the consistency of significant associations across different tests. Also, associations with these variables were determined in case of *Giardia* positivity in general, e.g. in case a sample was positive in at least one of the three tests (qPCR, IDEXX SNAP^®^ *Giardia* and DFA).

## Methods

### Dogs

Feces from 1291 dogs belonging to four groups (household dogs, shelter dogs, hunting dogs, and clinical dogs) were collected between October 2013 and December 2014 in the Netherlands [25]. The household dogs consisted of 551 privately owned dogs older than 6 months participating in a previous study on *T. canis* [26]. The dog owners collected the fecal samples and submitted them by mail. Moreover, the dog owners completed a questionnaire to provide relevant metadata about the sampled dogs, including age and sex.

The shelter dogs consisted of 278 dogs from 16 shelter-kennels, and the hunting dogs (scent hounds) consisted of 65 dogs from two hunting-kennels. Feces samples were collected by instructed personnel at the kennel or by veterinarians in training and, if available, information on sex and age was provided. The group clinical dogs consisted of 397 dogs with different underlying health conditions from which a fecal sample was submitted to the Veterinary Microbiological Diagnostic Center (VMDC) of the Faculty of Veterinary Medicine of Utrecht University for endoparasite testing. Most fecal samples were sent in to diagnose a possible parasitic cause of clinical symptoms and a few for control of therapy or for routine monitoring. Information on sex and age of these dogs was also provided in most cases. No age restriction was imposed on dogs in the shelter, hunting and clinical populations. The age of the dogs was classified as ≤ 6 months, 7–12 months, 1–2 years (13–24 months), 2–7 years (25–84 months) or > 7 years (> 85 months). All samples were collected and processed as described in Uiterwijk et al. [25].

### Fecal consistency score

Each fecal sample was scored for consistency. A fecal consistency classification system was developed, with classes ranging from 1 (liquid feces) to 7 (very hard, crumbly feces). For the calculations of association with fecal consistency, feces with consistency score 1 and 2 were considered loose stool and ≥ 3 were considered non-loose.

### Diagnostic techniques

Fecal samples were examined with four different techniques, as described in a previous study [25]. For detection of *Giardia* a qPCR, rapid enzyme immunochromatographic assay (IDEXX SNAP^®^ *Giardia*, IDEXX Laboratories Inc, Westbrook, Maine, USA) and direct immunofluorescence assay (DFA Merifluor *Cryptosporidium*/*Giardia* kit Meridian Bioscience Diagnostics Inc, Cincinnati, Ohio, USA) were used. For detection of gastrointestinal parasites, a centrifugal sedimentation and flotation technique (CSF) was performed. Coproscopical analysis with CSF involved examining all microscopic slides for presence of eggs, oocysts and cysts of gastrointestinal helminths and protozoa at 40×, 100× and 400× magnification. Identification was based on the reference manual issued by the AAVP [27]. Oocysts could either not be specifically determined as *Cystoisospora* sp. or *Eimeria* sp. or clearly identified to belong to one of these genera. Oocysts that could not be determined clearly, were categorized as coccidia. Presence of *Eimeria* sp. oocysts was considered as proof of coprophagy, as *Eimeria* spp. are non-canid gastrointestinal parasites.

Half of the 1291 collected samples (*n* = 646; 275 household dogs, 137 shelter dogs, 34 hunting dogs and 200 clinical dogs) were randomly selected and tested with qPCR. Results of these 646 samples were used for the latent class analysis published earlier [25] and for association analysis of the qPCR and *Giardia* positivity results in the present paper.

### Statistical analysis

Associations were investigated based on the outcome of each diagnostic test separately, i.e. qPCR, IDEXX SNAP^®^ *Giardia*, and DFA, and of *Giardia* positivity in general (e.g. positive with at least one of the abovementioned three tests). The outcome of CSF was not used in the association analysis because of its low sensitivity in one-day samples [25]. For binary (positive/negative) outcome variables, associations were investigated using multivariable logistic regression analysis and expressed as adjusted odds ratios (OR) and corresponding 95% confidence intervals (95% CI). The variables age group (≤ 6 months, 7–12 months, 1–2 years, 2–7 years, and > 7 years), sex (male or female), neuter status (neutered or entire), dog’s group of origin (household dogs, shelter dogs, hunting dogs, or clinical dogs), and fecal consistency (loose or non-loose) were always controlled for in the analysis by including them as covariates in the logistic regression models. However, no information on sex and age was available for the hunting dogs, so these variables could not be studied for these dogs.

Associations between cysts per gram (CPG) and semi-quantitative detection of cyst shedding by CSF were assessed using the Chi-square, Fisher’s exact, or two-sample Wilcoxon rank-sum test, as appropriate. The relationship between the quantitative outcome of the qPCR and age group and sex were assessed using negative binominal regression, with associations being expressed as incidence rate ratios (IRR) and corresponding 95% CIs. The relationship between CPG and fecal consistency, as well as between CPG and the presence of other gastrointestinal parasites excluding *Eimeria*, was assessed using Kruskal–Wallis rank test, while the relationship between semi-quantitative cyst detection with CSF and fecal consistency, or with the presence of other gastrointestinal parasites excluding *Eimeria*, was assessed using the Chi-square. A two-sample Wilcoxon rank-sum (Mann-Whitney) test was used to assess differences in median age between dogs with or without loose stool. In all analyses, a cluster-correlated robust variance estimator [28] was included to account for non-independency of observations from dogs living in the same environment, such as the same household or kennel. A maximum of 755 clusters were present in the whole data set. Statistical analysis was performed using STATA 13 (StataCorp LP, College Station, USA).

## Results

### Descriptive statistics

Of the total 1291 dogs, age information was available for 1183 dogs: household dogs (*n* = 547); shelter dogs (*n* = 247); and clinical dogs (*n* = 389). The overall median age was 4.2 years (interquartile range, IQR: 2.2–7.3 years). Within the different dog groups, there were significant differences in age distribution (*χ*^2^ = 78.4, *df* = 2, *P* = 0.0001). Median age was 4.4 years (IQR: 3.0–8.1 years) among household dogs, 4.6 years (IQR: 2.1–7.0 years) among shelter dogs, and 2.8 years (IQR: 0.5–6.0 years) among clinical dogs. For the hunting dogs, detailed information about age was not available, but they were all older than 6 months.

Information about sex and neuter status was available for 1127 dogs: household dogs (*n* = 546); shelter dogs (*n* = 223); and clinical dogs (*n* = 358). Overall, sexes were equally distributed, with a male-to-female (M:F) ratio of 1.06. However, between dog groups, differences were observed. M:F ratio for household dogs was 0.72, for shelter dogs 1.9, and for clinical dogs 1.3. In total, 423 dogs were neutered, of which 46.9% were female and 28.7% male. For 165 dogs, including all hunting dogs, no sex or neuter information was available.

The mean size of the kennels was 28.3 dogs (range 7–73), of which on average 19.1 dogs (range 7–38) were sampled. In the two hunting dog kennels, respectively 70 and 73 dogs were present (sample size 32 and 33, respectively), and in the shelter-kennels on average 22.9 dogs were present (range 7–70). In the shelter-kennels, on average 17.4 dogs per kennel were sampled (range 7–38). Of the total number of 18 kennels, 15 (83.3%) had at least one *Giardia*-positive dog (determined with qPCR).

### Samples examined with each diagnostic test

Of the total 1291 samples, 646 were tested with qPCR (189 positives; 29.3%, 95% CI: 23.7–35.5%), 1154 were tested with IDEXX SNAP^®^ *Giardia* test (198 positives; 17.2%, 95% CI: 14.2–20.6%) and 1288 were tested with DFA (243 positives; 18.9%, 95% CI: 16.0–21.9%). Of the 1274 samples tested with CSF, 141 were *Giardia*-positive (11.1%, 95% CI: 8.1–15.0%). Of the 646 samples that were tested with qPCR, IDEXX SNAP^®^ *Giardia* and DFA, 207 samples (32.0%, 95% CI: 26.6–38.0%) were *Giardia-*positive for at least one of the three tests. Overall, 573 samples were examined with all four tests. Additional file 1: Table S1 and Additional file 2: Table S2 show the prevalence of gastrointestinal parasites over dog groups and age, respectively. Raw data for all samples are provided in Additional file 3: Table S3.

### Fecal consistency

Fecal consistency scores (FCS) were determined for 1253 samples. In total, 283 dogs (22.6%, 95% CI: 20.3–25.0%) had loose stool. Loose stool was detected most often in hunting dogs (64.6%, 95% CI: 51.8–76.1%), followed by clinical dogs (38.6%, 95% CI: 33.6–43.8%), shelter dogs (17.0%, 95% CI: 12.7–21.9%) and household dogs (9.6%, 95% CI: 7.2–12.4%). Hunting dogs (OR: 17.2, 95% CI: 4.7–62.8, *P* < 0.0001) and clinical dogs (OR: 5.9, 95% CI: 4.0–8.8, *P* < 0.0001) had significantly more often loose stool compared to household dogs. Dogs with loose stool had a significant lower median age (3.2 years, IQR: 0.9–6.1 years) than dogs with no loose stool (4.3 years, IQR: 2.3–7.3 years) (Wilcoxon rank-sum test, *Z* = − 4.6, *P* < 0.00001).

### Association results

Table 1 shows the results from the association analysis for qPCR, IDEXX SNAP^®^ *Giardia*, DFA, and *Giardia*-positivity in general (e.g. *Giardia*-positive sample with qPCR, IDEXX SNAP^®^ *Giardia* and/or DFA). With qPCR, a higher *G. duodenalis* prevalence for all examined variables was found compared to IDEXX SNAP *Giardia*® and DFA, except for the presence of other gastrointestinal parasites. In samples also diagnosed positive for other gastrointestinal parasites, the prevalence of *G. duodenalis* was more comparable between the three tests.

**Table 1:** Prevalence and associations of *G. duodenalis* presence, determined with qPCR, IDEXX SNAP® *Giardia* and DFA, for gastrointestinal parasites, dog population, age groups, sex, neuter status and fecal consistency

	qPCR% (95% CI)	OR (95% CI)	SNAP% (95% CI)	OR (95% CI)	DFA% (95% CI)	OR (95% CI)	*Giardia*-positive (95% CI)	OR (95% CI)
Parasites								
Any helminth	22.5 (9.2–45.4)	1.32 (0.52–3.39) *P* = 0.560	22.1 (10.1–41.5)	**2.29** (1.08–4.83) *P* = 0.030	20.1 (9.4–38.0)	**2.15** (1.10–4.22) *P* = 0.026	22.0 (9.5–43.1)	1.91 (0.78–4.71) *P* = 0.158
*Toxocara* sp.	6.4 (3.9–10.5)	2.08 (0.75–5.81) *P* = 0.159	5.6 (3.0–10.3)	1.89 (0.86–4.12) *P* = 0.111	6.7 (4.0–11.0)	**2.80** (1.34–5.86) *P* = 0.006	6.8 (4.2–10.8)	2.67 (0.95–7.48 *P* = 0.061
*Toxascaris leonina*	7.0 (1.7–24.4)	0.76 (0.74–7.83) *P* = 0.820	6.7 (2.1–19.8)	**5.77** (2.21–15.02) *P* = 0.0001	5.4 (1.7–15.7)	2.01 (0.35–11.39) *P* = 0.430	6.3 (1.5–22.7)	0.67 (0.62–7.31) *P* = 0.746
*Trichuris* sp.^a^	16.6 (4.9–43.2)	**8.70** (3.63–20.86) *P* = 0.0001	14.9 (4.6–39.0)	**2.56** (1.40–4.70) *P* = 0.002	12.6 (3.5–36.1)	**2.48** (1.03–5.9) *P* = 0.043	15.6 (4.8–40.2)	**8.96** (4.04–19.86) *P* = 0.0001
Strongyle type eggs	16.6 (4.7–44.6)	0.84 (0.09–7.91) *P* = 0.876	14.9 (4.6–39.0)	3.38 (0.56–20.33) *P* = 0.184	12.6 (3.7–34.6)	1.38 (0.28–6.67) *P* = 0.690	15.1 (4.2–41.8)	0.66 (0.69–6.32) *P* = 0.720
*Taenia/Echinococcus* sp.	0.5 (0.09–3.2)	nc	0	nc	0	nc	0.5 (0.07–2.9)	nc
Any protozoa	11.8 (4.4–28.0)	1.61 (0.77–3.35) *P* = 0.206	10.3 (4.8–20.9)	1.29 (0.51–3.24) *P* = 0.586	9.7 (5.4–16.6)	1.61 (0.77–3.35) *P* = 0.206	10.8 (4.0–26.0)	1.09 (0.42–2.85) *P* = 0.865
Coccidia/C*ysto-isospora* sp.	4.3 (2.0–8.8)	1.60 (0.49–5.23) *P* = 0.434	5.7 (3.1–10.3)	1.30 (0.47–3.57) *P* = 0.614	6.7 (4.0–11.1)	1.71 (0.77–3.80) *P* = 0.190	3.9 (1.9–8.0)	1.41 (0.45–4.39) *P* = 0.558
*Eimeria* sp.	8.6 (2.2–28.4)	1.28 (0.26–6.26) *P* = 0.763	5.6 (2.0–15.0)	2.38 (0.54–10.52) *P* = 0.255	4.6 (1.8–11.1)	**3.48** (1.25–9.66) *P* = 0.017	7.8 (2.0–26.5)	1.11 (0.24–5.16) *P* = 0.898
Any parasite	27.3 (13.4–47.6)	1.32 (0.64–2.73) *P* = 0.446	27.7 (15.3–44.8)	**1.92** (1.01–3.68) *P* = 0.048	26.4 (15.1–42.0)	**1.95** (1.17–3.24) *P* = 0.011	26.3 (13.4–45.3)	1.58 (0.79–3.16) *P* = 0.198
Any parasite excl. *Eimeria*	26.2 (12.4–47.2)	1.38 (0.64–2.97) *P* = 0.411	26.7 (14.2–44.4)	1.93 (0.99–3.75) *P* = 0.054	25.9 (14.7–41.7)	**1.96** (1.17–3.30) *P* = 0.011	25.4 (12.4–44.9)	1.68 (0.81–3.50) *P* = 0.167
Dog population								
Household	17.1 (12.9–22.3)	Ref	5.3 (3.5–7.9)	Ref	11.6 (9.1–14.7)	Ref	21.5 (16.8–27.0)	Ref
Group housed^b^	45.6 (30.6–61.4)	**3.77** (1.93–7.35) *P* = 0.0001	25.1 (18.4–33.2)	**5.52** (3.05–9.96) *P* = 0.0001	25.1 (18.1–33.6)	**2.32** (1.43–3.78) *P* = 0.001	48.0 (34.0–62.3)	**3.12** (1.70–5.70) *P* = 0.0001
Shelter	35.0 (24.1–47.8)	**1.95** (1.07–3.55) *P* = 0.030	21.6 (15.1–29.9)	**3.22** (1.72–6.00) *P* = 0.0001	20.9 (15.4–27.6)	**2.00** (1.10–3.65) *P* = 0.024	38.0 (28.0–49.1)	1.63 (0.95–2.80) *P* = 0.074
Hunting^b^	88.2 (83.2–91.9)	**32.60** (17.23–61.59) *P* = 0.0001	40.0 (31.1–49.7)	**9.87** (4.88–19.98) *P* = 0.0001	43.1 (26.6–61.2)	**4.74** (1.93–11.64) *P* = 0.001	88.2 (83.2–92.0)	**23.94** (12.67–45.22) *P* = 0.0001
Clinical	32.0 (25.8–38.8)	1.14 (0.58–2.25) *P* = 0.709	22.8 (18.9–27.3)	**2.30** (1.22–4.33) *P* = 0.010	23.4 (19.4–27.8)	0.80 (0.45–1.30) *P* = 0.322	33.0 (26.8–39.9)	0.88 (0.46–1.68) *P* = 0.703
Age group								
≤ 6 months	56.0 (41.9–69.2)	Ref	46.2 (36.7–55.9)	Ref	49.0 (39.7–58.5)	Ref	56.0 (41.9–69.2)	Ref
7–12 months	54.3 (37.5–70.2)	1.27 (0.49–3.33) *P* = 0.624	32.9 (22.3–45.5)	**0.55** (0.27–1.13) *P* = 0.102	35.2 (24.9–47.1)	0.52 (0.26–1.04) *P* = 0.065	57.1 (40.2–72.6)	1.36 (0.53–3.56) *P* = 0.526
1 year	30.2 (18.0–46.1)	**0.30** (0.11–0.81) *P* = 0.018	14.3 (7.1–26.8)	**0.15** (0.06–0.37) *P* = 0.0001	17.2 (10.7–26.6)	**0.19** (0.08–0.43) *P* = 0.0001	32.6 (20.0–48.4)	**0.33** (0.53–0.88) *P* = 0.028
2–7 years	23.0 (17.9–29.0)	**0.26** (0.11–0.63) *P* = 0.003	12.4 (9.4–16.2)	**0.25** (0.13–0.49) *P* = 0.0001	14.1 (11.3–17.6)	**0.17** (0.92–0.32) *P* = 0.0001	25.3 (20.1–31.4)	**0.27** (0.12–0.64) *P* = 0.003
> 7 years	16.4 (11.3–23.2)	**0.22** (0.09–0.57) *P* = 0.002	8.3 (5.2–13.0)	**0.17** (0.08–0.38) *P* = 0.0001	10.1 (7.2–14.0)	**0.13** (0.06–0.25) *P* = 0.0001	21.2 (15.5–28.4)	**0.29** (0.12–0.72) *P* = 0.008
Sex								
Female	25.3 (20.3–31.0)	Ref	12.6 (9.9–16.0)	Ref	15.0 (12.2–18.3)	Ref	27.8 (22.6–33.5)	Ref
Male	23.3 (18.5–28.9)	0.77 (0.50–1.18) *P* = 0.229	16.3 (13.1–20.0)	1.24 (0.85–1.81) *P* = 0.269	17.7 (14.4–21.4)	1.17 (0.83–1.64) *P* = 0.382	27.1 (22.0–32.8)	0.87 (0.57–1.31) *P* = 0.501
Neuter status								
Female intact	37.0 (29.8–44.9)	**3.28** (1.60–6.72) *P* = 0.001	18.6 (14.3–23.9)	1.85 (0.89–3.88) *P* = 0.101	19.2 (15.1–24.2)	1.10 (0.64–1.88) *P* = 0.251	38.3 (30.9–46.3)	**2.58** (1.32–5.02) *P* = 0.005
Female neutered	11.0 (6.6–17.8)	Ref	5.3 (3.0–9.3)	Ref	10.2 (7.0–14.6)	Ref	15.0 (9.7–22.4)	Ref
Male intact	25.4 (19.6–32.1)	1.72 (0.86–3.43) *P* = 0.124	19.7 (15.7–24.4)	**2.20** (1.07–4.54) *P* = 0.033	20.9 (17.0–25.4)	1.37 (0.80–2.34) *P* = 0.672	30.8 (24.9–37.5)	1.77 (0.94–3.34) *P* = 0.079
Male neutered	18.4 (11.6–27.8)	1.69 (0.74–3.84) *P* = 0.210	7.9 (4.5–13.4)	1.17 (0.48–2.83) *P* = 0.732	9.6 (5.7–16.0)	0.86 (0.42–1.74) *P* = 0.251	18.4 (11.6–27.8)	1.21 (0.57–2.59) *P* = 0.620
Fecal consistency								
Loose	40.8 (27.4–55.8)	1.31 (0.77–2.23) *P* = 0.326	26.5 (21.8–31.8)	1.42 (0.90–2.23) *P* = 0.269	27.4 (22.0–33.6)	1.49 (0.94–2.39) *P* = 0.093	43.7 (30.5–57.8)	1.41 (0.84–2.34) *P* = 0.192
Non-loose	25.9 (21.1–31.4)	Ref	14.3 (11.2–18.2)	Ref	16.3 (13.6–19.4)	Ref	28.9 (24.1–34.1)	Ref
Total	29.3 (23.7–35.5)		17.2 (14.2–20.6)		18.8 (16.0–22.0)		32.0 (26.7–38.0)	

^a^OR adjusted for fecal consistency and clustering

^b^OR adjusted for fecal consistency, clustering and dog population

*Abbreviations*: OR, odds ratio; 95% CI, 95% confidence interval; *P*, *P*-value; SNAP, IDEXX SNAP® *Giardia*, Ref, reference group; nc, not calculable

*Notes*: OR adjusted for dog population, age, sex, neuter status, fecal consistency score and clustering. *Giardia* positive: positive with at least one of the three tests

*G. duodenalis* prevalence in %. OR in bold: significant association (*P* < 0.05)

Significant associations with *Giardia* positivity over the three diagnostic tests were found for dog group and age group. Group-housed kenneled dogs, especially the hunting dogs, were significantly more often *Giardia-*positive than the household dogs. Dogs older than 12 months were significantly less often *Giardia-*positive than younger dogs.

Significant differences in prevalence between household dogs and clinical dogs were not consistent across tests. Significant associations of *Giardia* presence with presence of gastrointestinal parasites were mostly found when IDEXX SNAP^®^ *Giardia* or DFA were used. Only for *Trichuris*, a significant association with *G. duodenalis* was found irrespectively of the test used. Overall, there were no significant associations between having loose stool and positivity for *G. duodenalis* with any of the three diagnostic tests. However, within the group of the household dogs, there was a consistent and significant association of *G. duodenalis* positivity and loose stool (Table 2). The prevalence and associations for *Giardia* positivity were, overall, comparable with the prevalence and associations as observed with the qPCR.

**Table 2:** Associations of qPCR, IDEXX SNAP® *Giardia*, DFA and *Giardia* positivity with fecal consistency for dog population

	qPCR OR (95% CI)	SNAP OR (95% CI)	DFA OR (95% CI)	*Giardia*-positive OR (95% CI)
Household				
Loose	**2.50** (1.05–5.96) *P* = 0.038	**5.77** (2.14–15.58) *P* = 0.001	**2.71** (1.31–5.62) *P* = 0.007	**2.83** (1.27–6.32) *P* = 0.011
Non-loose	Ref	Ref	Ref	Ref
Group housed^a^				
Loose	1.64 (0.94–2.86) *P* = 0.083	**1.57** (1.11–2.22) *P* = 0.012	1.39 (0.41–4.69) *P* = 0.596	1.66 (0.98–2.82) *P* = 0.058
Non-loose	Ref	Ref		Ref
Shelter				
Loose	2.00 (0.65–6.23) *P* = 0.228	0.76 (0.32–1.78) *P* = 0.522	1.64 (0.63–4.31) *P* = 0.312	2.16 (0.78–6.00) *P* = 0.137
Non-loose	Ref	Ref	Ref	Ref
Hunting^a^				
Loose	**2.33** (1.99–2.73) *P* = 0.0001	**0.61** (0.53–0.70) *P* = 0.0001	0.56 (0.30–1.06) *P* = 0.079	**2.33** (1.99–2.73) *P* = 0.0001
Non-loose	Ref	Ref	Ref	Ref
Clinical				
Loose	0.71 (0.32–1.57) *P* = 0.393	1.14 (0.64–2.06) *P* = 0.656	0.92 (0.52–1.63) *P* = 0.773	0.64 (0.29–1.45) *P* = 0.287
Non-loose	Ref	Ref	Ref	Ref

^a^OR adjusted for fecal consistency, clustering and dog population

*Abbreviations*: OR, odds ratio; 95% CI, 95% confidence interval; *P*, *P*-value; SNAP, IDEXX SNAP® *Giardia*; Ref, reference group

*Notes*: OR adjusted for dog population, age, sex, neuter status, fecal consistency score and clustering. *Giardia* positive: positive with at least one of the three tests. OR in bold: significant association (*P* < 0.05)

### Cysts per gram (CPG) and semi-quantitative cyst detection

There was a significant difference in CPG shed determined with qPCR by dogs of different age groups (*χ*^2^ = 13.1, *df* = 4, *P* = 0.0108). The 28 positive dogs up to 6 months of age showed highest CPG (median 2.7 × 10^4^; IQR 5.8 × 10^3^–1.0 × 10^5^) and between 2 and 7 years the lowest (median 4.8 × 10^3^; IQR 1.5 × 10^3^–2.0 × 10^4^).

The CPG in qPCR-positive dogs with loose stool (median CPG 1.1 × 10^4^; IQR 3.6 × 10^3^–4.5 × 10^4^) was not significantly higher than in qPCR positive dogs with no loose stool (median CPG 6.8 × 10^3^; IQR 1.5 × 10^3^–2.7 × 10^4^). Moreover, there was no significant association between CPG and sex and between CPG and presence of other canine gastrointestinal parasites.

With semi-quantitative cyst detection determined with CSF, there was no significant difference between the number of cysts detected in dogs with loose stool or in dogs with no loose stool. When other canine gastrointestinal parasites were present, significantly less cysts were detected with semi-quantitative cyst detection (*χ*^2^ = 10.05, *df* = 3, *P* = 0.018).

## Discussion

We determined several host correlates of *G. duodenalis* infection in dogs as determined by three commonly used diagnostic tests separately (qPCR, IDEXX SNAP^®^ *Giardia* and DFA) and the results of the three tests combined (*Giardia*-positive). Correlations with host-related factors were assessed for the three tests separately, to determine whether associations were independent of the diagnostic test used. Consistent positive associations with the presence of *Giardia* were found for group-housed dogs overall and hunting dogs, and consistent negative associations were found for dogs older than one year of age. This is in accordance with previous reports [29–31]. There were no significant associations between the presence of *G. duodenalis* and any other gastrointestinal parasite, except for a positive association with *Trichuris* sp. This can be explained by the fact that *Trichuris* sp. prevalence in hunting dogs was very high (98.5%, see Additional file 1: Table S1), compared to other dog groups. When the hunting dogs were excluded from the analysis, there was no significant association anymore (data not shown).

When other gastrointestinal parasite eggs or oocysts were present, significantly fewer *Giardia* cysts (semi-quantitatively determined with CSF) were detected, but there was no significant association with CPG (determined with qPCR). Because *Giardia* cysts are small and lucent, they can be easily missed, especially when larger eggs and oocysts are present. Moreover, cysts are present at ‘a slightly different flotation height’ than eggs and oocysts and can therefore more easily be out of focus and consequently be missed. With molecular or immunological detection, there is no such disadvantage. Although staff and trainees were trained and aware about this, it might account for the difference in findings between CSF and qPCR. Noteworthy is that prevalence of *G. duodenalis* was found to be much more similar between qPCR, IDEXX SNAP^®^ *Giardia*, DFA and *Giardia* positivity when other gastrointestinal parasites were present. However, the prevalence of *G. duodenalis* measured with qPCR was much higher compared to the other two tests when associations with all other variables were examined. We cannot fully explain this finding. The selection of samples for qPCR testing was done randomly, so bias towards samples in which other gastrointestinal parasites were present seems unlikely. In the samples in which gastrointestinal parasites were present, relatively greater amounts of *Giardia* cyst wall antigens and cysts were present, leading to more positive results with IDEXX SNAP^®^ *Giardia* and DFA, respectively. The finding of more significant associations between gastrointestinal parasites and the presence of *Giardia* with IDEXX SNAP^®^ *Giardia* and DFA would support this. However, this contrasts with the absence of a significant association of CPG (detected with qPCR) and the presence of gastrointestinal parasites.

In the present study, fecal consistency was scored, because diarrhea or loose stool is a symptom of giardiosis. Overall, the intensity of shedding of *Giardia* cysts, determined with both qPCR and CSF, was not significantly higher in dogs with loose stool. Moreover, the difference between *G. duodenalis*-positive dogs with loose stool and *G. duodenalis*-positive dogs with no loose stool was not significant. This finding was independent of the test (qPCR, IDEXX SNAP^®^ *Giardia*, DFA separately and combined results) used. Remarkably, within the household dogs, which were all older than six months, there was a significant positive association between *G. duodenalis* presence and loose stool. Prevalence of *G. duodenalis* and the number of dogs with loose stool were the lowest in the household dogs. Thus, household dogs appear to have a relatively small chance of being infected with *G. duodenalis*, but when they do, they seem more prone to develop symptoms of giardiosis (loose stool). For the clinical dogs, in contrast to what might be expected, there was also no significant association with loose stool. Of note, the fecal samples in the clinical dog group were sent to the VMDC for endoparasitic examination for various reasons, not only because of diarrhoea.

Šlapeta et al. [4] reported that *G. duodenalis* has replaced hookworm and roundworm in domestic dogs. Other studies found that *G. duodenalis* was significantly more often detected after anthelmintic treatment, both in humans [17, 20] and in dogs [11]. Consequently, it can be hypothesized that *G. duodenalis* filled a niche in the gut left by previously present gastrointestinal parasites or shaped the immune response and/or gut microbiome in detriment for other gastrointestinal parasites (or *vice versa*). A recent American study comparing prevalence of gastrointestinal parasites during 1984–1991 to that of a period almost two decades later (2000–2007), showed a trend with decreasing helminth prevalence and increasing *G. duodenalis* prevalence [32]. Similar findings were obtained in Germany [33, 34]. Comparing previously performed studies in Dutch dogs (sample period 1972–2012) with our results, taking into account diagnostic techniques and dog populations, does not give uniform outcomes [26, 35–39] (see Additional file 3: Table S4). In household dogs, the prevalence of nematodes (3.7% in 1994–1995 to 3.3% in our study) and helminths (8.1% in 2011–2012 to 4.7% in our study) seems to have declined slightly, but the prevalence of *T. canis* is more variable over the years (2.9% in 1994–1995 and 4.4% in 2007 to 3.1% in our study) [35, 37]. *Giardia* was only tested in household dogs in one Dutch study and compared to that study the prevalence has decreased (15.2% in 2007 to 5.3% in our study) [37]. The prevalence of nematodes in shelter dogs decreased (e.g. nematodes 16.1% in 2001 to 9.3% in our study), compared to the study by Le Nobel et al. [38]. Another study has determined nematode prevalence in dogs from breeding kennels [36]. Because in breeding kennels more young dogs are present than in shelter-kennels, it is difficult to fully compare our results with that study. Nevertheless, when comparing prevalences in adult shelter dogs with adult breeding dogs, a decrease in nematode prevalence can be seen, especially for *T. vulpis* (11% in 1993 to 1.4% in our study) (see Additional file 3: Table S4). *Giardia* was not tested in the previous studies, so we cannot compare our *Giardia* results in shelter dogs over the years. Also, clinical dogs were not studied in The Netherlands previous to our study.

Comparing our results with a study performed (sampling period 2004–2007) in a neighbouring country, Belgium, reveal that the prevalence of nematodes decreased and *Giardia* prevalence increased in the two best comparable dog populations (household dogs and clinical dogs) [29]. In the Belgian household dogs, the prevalence of *T. canis* for example was 4.4%, compared to 3.1% in our study, and of *Giardia* 9.3%, compared to 11.6% in our study. The prevalence of *T. canis* in the Belgian clinical dogs was 7.4%, compared to 4.2% in our study and the prevalence of *Giardia* was 18.1%, compared to 23.4% in our study. However, based on the available data, no informed statements about the course of prevalence over the years are possible.

## Conclusions

*Giardia* is the most prevalent gastrointestinal parasite in household, shelter and clinical dogs. Although *Giardia* prevalence is also high in hunting dogs, *Trichuris* and strongyle-type eggs are most prevalent. *Giardia* infection does not necessarily lead to loose stool. Indeed, the association between loose stool and *Giardia* infection was only significant for household dogs. *Giardia* was also more often found in dogs younger than one year and in group-housed dogs (shelter and hunting dogs). For other variables, associations with *Giardia* positivity were not consistent over diagnostic tests. This indicates that certain associations may depend on test characteristics, and that a reported association based upon one diagnostic test should be interpreted with care. The prevalence of nematodes/helminths in dogs in the Netherlands and surrounding area varies over the last decades. For *Giardia* in dogs it is even more difficult to speculate about trends, as there are only a few studies in which *Giardia* was included for the region in question. The available information about occurrence of gastrointestinal parasites over time is insufficient to provide sound statements about the increase or decrease of the prevalence of helminths and *Giardia* in dogs.

## Supplementary information

**Additional file 1: Table S1.** Prevalence of gastrointestinal parasites for the different dog populations.

**Additional file 2: Table S2.** Prevalence of gastrointestinal parasites for the different age groups.

**Additional file 3: Table S3.** Raw data. **Table S4.** Prevalence of gastrointestinal parasites, compared to previous performed studies in similar dog populations.

## Data Availability

The datasets used and/or analysed during the present study are presented in the article and its additional files or are available from the corresponding author upon reasonable request.

## Abbreviations

CI: confidence interval
DFA: direct immunofluorescence
OR: odds ratio
*P*: *P*-value
qPCR: quantitative real time PCR
REF: reference group
SNAP: IDEXX SNAP *Giardia*®
