# Supplementary material for: Host factors associated with Giardia duodenalis infection in dogs across multiple diagnostic tests
Source: Parasit Vectors. 2019 Nov 21;12:556. doi: 10.1186/s13071-019-3810-3 (PMC6873540; doi:10.1186/s13071-019-3810-3)
Supplement: Supplementary file 1 — Additional file 1: Table S1. Prevalence of gastrointestinal parasites for the different dog populations. [file 13071_2019_3810_MOESM1_ESM.docx]

**Additional file 1: Table S1.** Prevalence in % and 95% confidence interval (in brackets) of gastrointestinal parasites for the different dog populations, determined with CSF.

*G. duodenalis* prevalence determined with qPCR.

| **Parasite** | **Overall** | **n** | **Household** | **n** | **Group-housed kenneled** | **n** | **Shelter** | **n** | **Hunting** | **n** | **Clinical** | **n** |
| --- | --- | --- | --- | --- | --- | --- | --- | --- | --- | --- | --- | --- |
| **Total parasite** | 43.6 (35.8-51.8) | 732 | 27.4 (22.4-33.2) | 299 | 64.1 (45.0-80.0) | 220 | 49.0 (37.7-60.5) | 155 | 100 (94.5-100)* | 65 | 45.1 (38.4-52.0) | 213 |
| **Total parasite (excl. *Giardia*)** | 14.1 (8.8-21.7) | 1274 | 7.1 (5.1-9.8) | 551 | 28.0 (12.1-52.4) | 343 | 11.1 (7.4-16.4) | 278 | 100 (94.5-100)* | 34 | 11.6 (8.7-15.3) | 380 |
| **Total parasite (excl. *Eimeria*)** | 43.0 (35.1-51.3) | 732 | 26.9 (21.8-32.6) | 299 | 63.5 (43.9-79.4) | 220 | 48.1 (36.4-59.9) | 155 | 100 (94.5-100)* | 34 | 44.6 (37.9-51.5) | 213 |
| **Total helminth** | 10.2 (5.3-18.7) | 1274 | 4.7 (3.2-7.0) | 551 | 24.7 (9.2-51.6) | 343 | 7.2 (4.1-12.2) | 278 | 100 (94.5-100)* | 65 | 5.3 (3.4-8.1) | 380 |
| ***Toxocara* sp.** | 4.2 (3.1-5.5) | 1274 | 3.1 (1.8-5.1) | 551 | 5.8 (3.6-9.3) | 343 | 6.1 (3.5-10.5) | 278 | 4.6 (2.9-7.1) | 65 | 4.2 (2.6-6.8) | 380 |
| ***Toxascaris leonina*** | 2.4 (0.7-7.7) | 1274 | 0.0 (0.0-0.7)* | 551 | 7.8 (2.2-24.9) | 343 | 1.1 (0.1-7.6) | 278 | 36.9 (16.8-63.0) | 65 | 0.8 (0.2-2.4) | 380 |
| ***Trichuris* sp.** | 5.7 (1.8-17.2) | 1274 | 0.4 (0.09-1.4) | 551 | 19.8 (5.7-50.4) | 343 | 1.4 (0.4-4.5) | 278 | 98.5 (94.1-99.6) | 65 | 0.8 (0.2-3.3) | 380 |
| **Strongyle type eggs** | 6.1 (2.0-17.1) | 1274 | 1.8 (1.0-3.3) | 551 | 19.2 (5.3-50.5) | 343 | 0.7 (0.1-5.1) | 278 | 98.5 (94.1-99.6) |  | 0.5 (0.1-2.1) | 380 |
| ***Taenia*/*Echinococcus* sp.** | 0.2 (0.04-0.6) | 1274 | 0.0 (0-0.7)* | 551 | 0.6 (0.2-1.9) | 343 | 0.4 (0.06-2.1) | 278 | 1.5 (0.4-5.9) | 65 | 0.0 (0-1.0)* | 380 |
| **Total protozoa** | 35.8 (29.3-42.7) | 732 | 21.2 (16.5-26.8) | 299 | 53.2 (36.3-69.5) | 220 | 41.4 (30.1-53.6) | 155 | 95.1 (92.5-96.8) | 34 | 40.0 (33.3-47.1) | 213 |
| **Total protozoa (excl. *Giardia*)** | 5.8 (3.4-9.7) | 1274 | 3.1 (1.8-5.2) | 551 | 9.9 (3.4-2.6) | 343 | 4.7 (2.7-7.9) | 278 | 32.3 (7.1-74.8) | 65 | 6.1 (4.0-9.1) | 380 |
| **Total protozoa (excl. *Eimeria*)** | 34.2 (28.4-40.5) | 732 | 20.2 (15.6-25.8) | 299 | 50.0 (34.5-65.5) | 220 | 39.6 (28.4-52.0) | 155 | 91.7 (91.0-92.3) | 34 | 39.5 (32.8-46.6) | 213 |
| **Coccidia/*Cysto-isospora* sp.** | 3.6 (2.6-5.0) | 1274 | 2.5 (1.4-4.5) | 551 | 3.5 (1.7-7.2) | 343 | 3.2 (1.4-7.4) | 278 | 4.6 (1.1-16.7) | 65 | 5.3 (3.4-8.1) | 380 |
| ***Eimeria* sp.** | 2.7 (1.0-6.9) | 1274 | 1.5 (0.7-3.1) | 551 | 6.7 (1.7-22.8) | 343 | 1.8 (0.8-4.1) | 278 | 27.7 (6.7-67.2) | 65 | 0.8 (0.3-2.4) | 380 |
| ***Giardia duodenalis*** | 29.3 (23.7-35.5) | 646 | 17.1 (12.9-22.3) | 275 | 45.6 (30.6-61.4) | 171 | 35.1 (24.1-47.8) | 137 | 88.2 (83.2-91.9) | 34 | 32.0 (25.9-38.9) | 200 |

n= number of observations

*One-sided, 97.5% confidence interval
