# Supplementary material for: Host factors associated with Giardia duodenalis infection in dogs across multiple diagnostic tests
Source: Parasit Vectors. 2019 Nov 21;12:556. doi: 10.1186/s13071-019-3810-3 (PMC6873540; doi:10.1186/s13071-019-3810-3)
Supplement: Supplementary file 2 — Additional file 2: Table S2. Prevalence of gastrointestinal parasites for the different age groups. [file 13071_2019_3810_MOESM2_ESM.docx]

**Additional file 2: Table S2.** Prevalence in % and 95% confidence interval (in brackets) of gastrointestinal parasites for the age groups, determined with CSF.

*G. duodenalis* prevalence determined with qPCR.

| **Parasite** | **≤ 6 months** | **n** | **7-12 months** | **n** | **1 year** | **n** | **2-7 years** | **n** | **> 7 years** | **n** |
| --- | --- | --- | --- | --- | --- | --- | --- | --- | --- | --- |
| **Total parasite** | 73.4 (61.2-82.9) | 64 | 60.0 (42.9-75.0) | 35 | 42.6 (29.4-56.8) | 47 | 32.6 (27.1-38.6) | 319 | 29.7 (22.2-38.5) | 175 |
| **Total parasite (excl. *Giardia*)** | 21.6 (14.6-30.7) | 102 | 11.3 (5.8-20.8) | 71 | 8.1 (4.3-14.7) | 87 | 7.2 (5.2-9.9) | 596 | 9.1 (6.0-13.4) | 309 |
| **Total parasite (excl. *Eimeria*)** | 73.4 (61.2-82.9) | 64 | 60.0 (42.9-75.0) | 35 | 42.6 (29.4-56.8) | 47 | 31.8 (26.3-37.8) | 319 | 28.7 (21.4-37.5) | 175 |
| **Total helminth** | 7.7 (3.9-14.7) | 102 | 5.6 (2.2-13.7) | 71 | 5.7 (2.5-12.5) | 87 | 4.1 (2.7-6.2) | 596 | 6.6 (3.9-10.9) | 309 |
| ***Toxocara* sp.** | 6.9 (3.3-13.7) | 102 | 4.2 (1.4-11.8) | 71 | 4.6 (1.8-11.0) | 87 | 2.9 (1.7-4.6) | 596 | 5.2 (2.7-9.6) | 309 |
| ***Toxascaris leonina*** | 2.0 (0.5-7.5) | 102 | 0 (0-5.1)* | 71 | 0 (0-4.2)* | 87 | 0 (0-0.6)* | 596 | 1.3 (0.3-5.8) | 309 |
| ***Trichuris* sp.** | 0 (0-3.6)* | 102 | 1.4 (0.2-9.5) | 71 | 0 (0-4.2)* | 87 | 0.8 (0.3-2.6) | 596 | 0.3 (0.05-2.2) | 309 |
| **Strongyle type eggs** | 1.0 (0.1-6.7) | 102 | 0 (0-5.1)* | 71 | 1.1 (0.2-7.8) | 87 | 1.0 (0.4-2.3) | 596 | 1.9 (0.8-4.7) | 309 |
| ***Taenia*/*Echinococcus* sp.** | 0 (0-3.6)* | 102 | 0 (0-5.1)* | 71 | 1.1 (0.3-6.2) | 87 | 0 (0-0.6)* | 596 | 0 (0-1.2)* | 309 |
| **Total protozoa** | 67.8 (54.8-78.5) | 64 | 57.1 (40.2-72.6) | 35 | 34.1 (21.6-49.3) | 47 | 28.3 (22.9-34.4) | 596 | 21.0 (14.8-28.7) | 309 |
| **Total protozoa (excl. *Eimeria*)** | 67.8 (54.8-78.5) | 59 | 57.1 (40.2-72.6) | 35 | 34.1 (21.6-49.3) | 44 | 27.1 (21.7-33.1) | 303 | 19.3 (13.7-26.4) | 166 |
| **Total protozoa (excl. *Giardia*)** | 13.7 (8.3-21.9) | 102 | 5.6 (2.1-14.4) | 71 | 2.3 (0.6-8.1) | 87 | 3.9 (2.5-5.8) | 596 | 2.6 (1.3-4.9) | 309 |
| **Coccidia/*Cysto-isospora* sp.** | 13.5 (8.1-21.5) | 102 | 4.2 (1.3-12.5) | 71 | 2.3 (0.6-8.1) | 87 | 3.0 (1.8-5.0) | 596 | 1.6 (0.7-3.7) | 309 |
| ***Eimeria* sp.** | 0 (0-3.6)* | 102 | 2.8 (0.7-10.8) | 71 | 0 (0-4.2)* | 87 | 1.5 (0.8-3.0) | 596 | 1.6 (0.7-3.7) | 309 |
| ***Giardia duodenalis*** | 56.0 (41.9-69.2) | 50 | 54.3 (37.5-70.2) | 35 | 30.2 (18.0-46.1) | 43 | 23.0 (17.9-29.0) | 296 | 16.4 (11.3-23.2) | 165 |

n= number of observations

*One-sided, 97.5% confidence interval
